# Diagnosis of Bilateral Bow Hunter’s Syndrome in an Adolescent Using Dynamic Cervical Magnetic Resonance Angiography: A Case Report

**DOI:** 10.2174/0115734056432014260120104650

**Published:** 2026-02-02

**Authors:** Weijia Chen, Yongjun Wu, Jiawu Fu, Haiman Mo, Wanxin Jie, Wenchuan Xian

**Affiliations:** 1 Department of Neurology, Affiliated Hospital of Guangdong Medical University, Zhanjiang 524001, China; 2 Department of Radiology, Affiliated Hospital of Guangdong Medical University, Zhanjiang 524001, China

**Keywords:** Bow hunter’s syndrome, Magnetic resonance angiography, Pediatrics, Rotational vertebral artery occlusion syndrome, Cervical disc herniation, Digital subtraction angiography

## Abstract

**Background:**

Bow hunter's syndrome (BHS), also known as rotational vertebral artery occlusion syndrome, is a hemodynamic disorder caused by mechanical compression of the vertebral artery during head rotation or hyperextension. This compression may lead to transient occlusion or significant stenosis, resulting in posterior circulation ischemia. BHS is relatively rare in clinical settings, and most reported cases occur in middle-aged or elderly patients. Its etiology is commonly associated with degenerative cervical conditions, such as osteophyte formation or disc herniation. In addition, the condition most often involves unilateral vertebral artery compromise.

**Case Presentation:**

In this study, we report a rare case of BHS in a 15-year-old adolescent without noticeable cervical degenerative changes who experienced recurrent, unexplained posterior circulation cerebral infarctions. Dynamic cervical magnetic resonance angiography (MRA) revealed significant compression of both vertebral arteries during head and neck rotation, confirming a diagnosis of bilateral BHS. This presentation differs from the conventional understanding that BHS predominantly affects adults and is typically unilateral, suggesting that the diagnosis should also be considered in young patients, even in the absence of typical cervical lesions.

**Conclusion:**

This study is the first report identifying bilateral BHS as the cause of recurrent posterior circulation infarction in a teenager using dynamic MRA. Although dynamic digital subtraction angiography remains the gold standard for diagnosis, this case highlights the practical value of dynamic MRA in diagnosing BHS.

## 1 INTRODUCTION

Bow Hunter’s Syndrome (BHS) is a rare cause of vertebrobasilar insufficiency that most commonly occurs at the craniocervical junction. It is characterized by posterior circulation ischemia triggered by head rotation, with common symptoms including dizziness and syncope [1]. The primary pathophysiology of BHS is mechanical compression of the vertebral artery (VA) during neck rotation or hyperextension, whichmayresultfromosteophytes,cervical disc herniation, hypertrophied muscles or ligaments, or dynamic hemodynamic abnormalities [2]. In some cases, repetitive shear stress on the arterial endothelium may also promote thromboembolism, further contributing to the development of BHS [1].

BHS mainly affects middle-aged and elderly individuals, and cases in adolescents are rare [3]. In younger patients, BHS is often associated with prior trauma or vigorous physical activity involving the head or neck, which may increase the susceptibility of the VAs to compression during cervical rotation or extension [3]. Bilateral BHS, in which symptoms are induced by head rotation to both sides, is even rarer.

Digital subtraction angiography (DSA) remains the gold standard for diagnosing BHS because it allows direct visualization of dynamic VA occlusion during head rotation [4]. However, its invasiveness, associated radiation exposure, and the need for sedation or general anesthesia limit its applicability in pediatric populations [5]. Transcranial Doppler ultrasound (TCD) is another widely used, noninvasive, and cost-effective diagnostic tool that can be performed at the bedside [2]. However, its diagnostic accuracy is highly operator-dependent.

In this study, we report a rare case of a 15-year-old male with recurrent posterior-circulation strokes who was ultimately diagnosed with bilateral BHS by dynamic cervical magnetic resonance angiography (MRA). This case highlights the diagnostic value of dynamic MRA as a noninvasive, effective alternative for diagnosing BHS, particularly in pediatric patients.

## 2 CASE PRESENTATION

A 15-year-old male was admitted to the Department of Neurology, Affiliated Hospital of Guangdong Medical University, with a two-month history of recurrent dizziness and symptom relapse nine hours before admission. His medical history included thalassemia, with no hypertension, diabetes mellitus, or cardiovascular disease. He was physically active and played basketball regularly. The patient denied frequent neck massage.

**Fig. (1):**
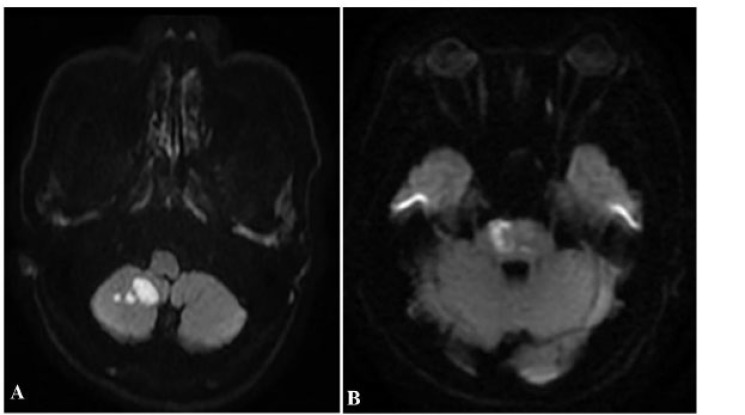
Brain MRI. Acute infarction in the (**A**) right cerebellar hemisphere and (**B**) right pons.

**Fig. (2):**
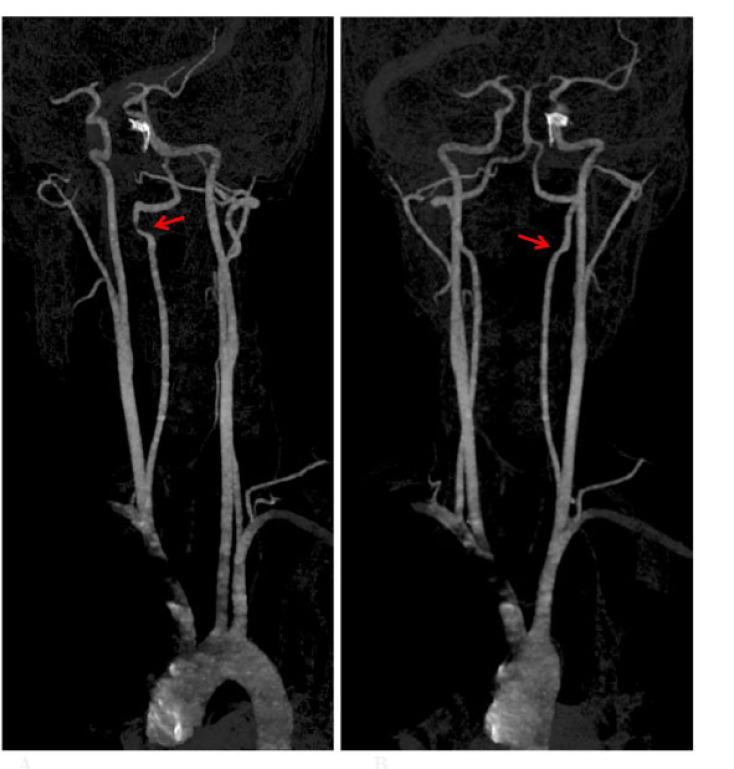
Cervical CTA. (**A**) Moderate stenosis of the right VA at the V3 segment. (**B**) Mild stenosis of the left VA at the V3 segment.

**Fig. (3):**
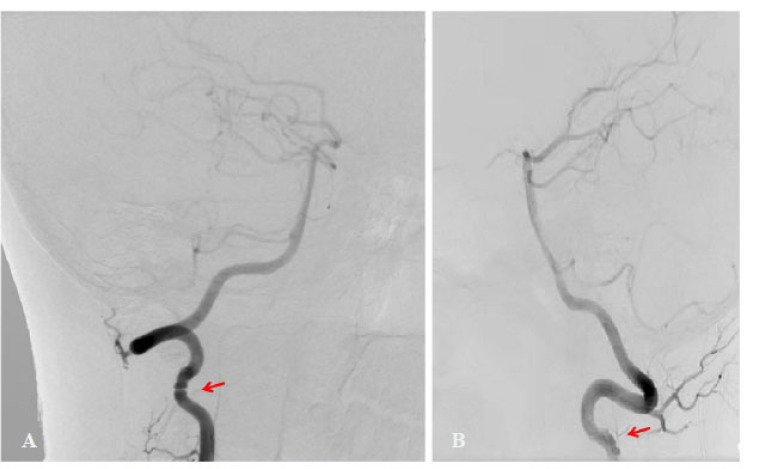
Cervical DSA. (**A**-**B**) Possible dissection of the right VA at the V3 segment.

Approximately two months before admission, the patient experienced sudden vertigo during a basketball game. The sensation was rotational, accompanied by nausea, vomiting, and occipital headache. Within minutes, he developed left-sided limb weakness, clumsiness, dysphagia with choking on liquids, and dysarthria. He was evaluated at a local hospital, where brain magnetic resonance imaging (MRI) (Fig. **1**), head and neck computed tomography angiography (CTA) (Fig. **2**), and DSA (Fig. **3**) were performed. These examinations revealed an acute cerebral infarction and a possible right VA dissection, although the underlying cause remained unclear. The patient was treated with aspirin (100 mg/day) and atorvastatin (20 mg/day), after which his symptoms gradually improved.

Nine hours prior to the current admission, the patient had a recurrence of the same symptoms while lifting his head during sweeping. MRI at a local emergency department revealed new infarcts in the right cerebellum and occipital lobe, leading to his referral to our hospital for further evaluation and management.

Upon admission, the patient was conscious and able to speak clearly, with a blood pressure of 120/70 mmHg. Horizontal nystagmus was observed bilaterally. The left nasolabial fold was flattened. He exhibited dysphagia with choking when drinking. Other cranial nerve examinations were unremarkable. Muscle strength was graded 5/5 in all four limbs. Bilateral Babinski signs were negative. No abnormalities were detected in deep or superficial sensation or coordination tests.

Laboratory investigations, including a complete blood count, serum biochemical panel, thrombophilia screening, antiphospholipid panels, comprehensive immunological profile, erythrocyte sedimentation rate, thyroid function tests, anti-nuclear and anti-neutrophil cytoplasmic antibodies, uric acid, homocysteine, lactate concentrations, and a full tumor-marker panel, were all within reference limits. TCD bubble study and 24-h dynamic electrocardiogram monitoring were likewise unremarkable.

**Fig. (4):**
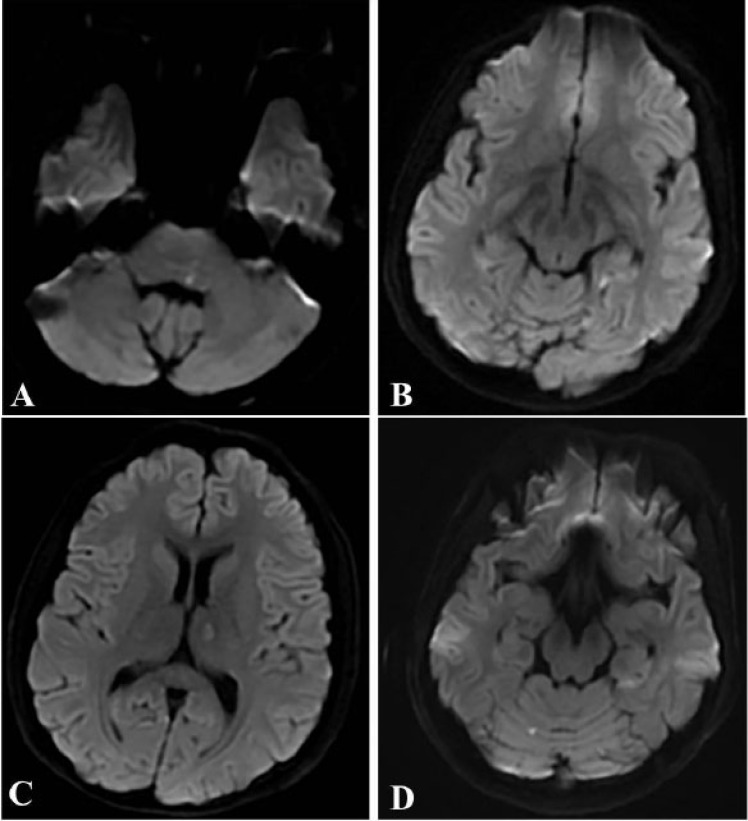
Brain MRI. Acute infarction in the (**A**) left pons; (**B**) occipital lobe; (**C**) thalamus; and (**D**) right cerebellum.

**Fig. (5):**
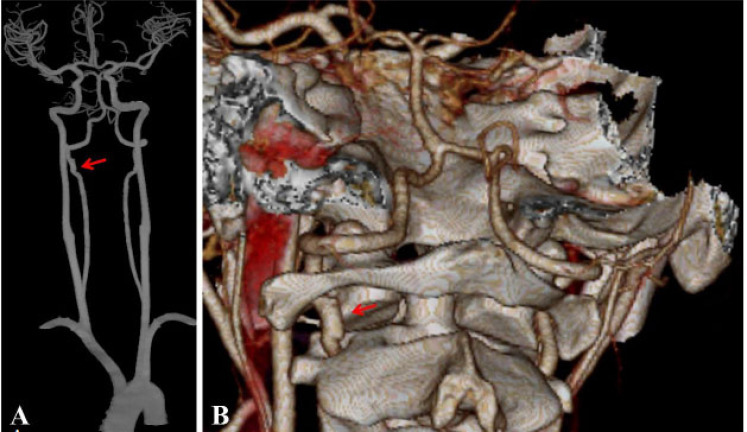
Cervical CTA. (**A**-**B**) Possible dissecting aneurysm of the right VA at the V3 segment.

Brain MRI (Fig. **4**) demonstrated acute infarctions in the left pons, occipital lobe, thalamus, and right cerebellum. Head and neck CTA suggested a possible aneurysm in the V3 segment of the right VA (Fig. **5**).

**Fig. (6):**
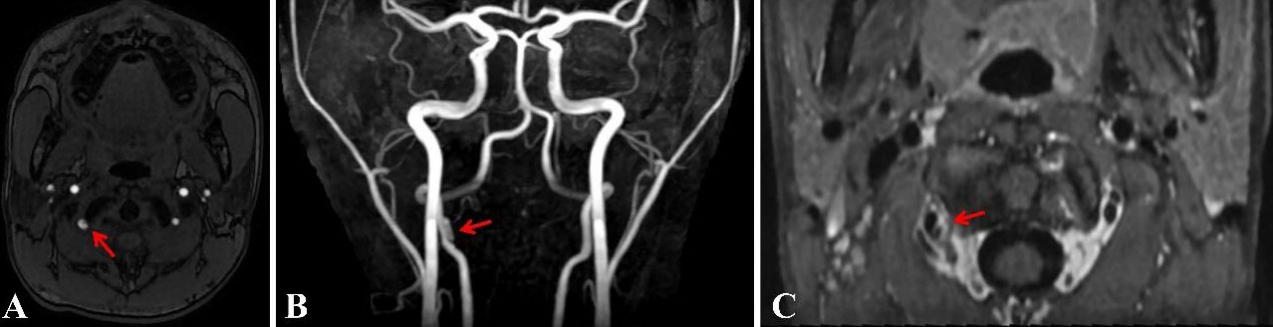
(**A**-**B**) A dissecting aneurysm in the right VA (V3 segment); (**C**) Thrombosis within the false lumen demonstrated on high-resolution MRI.

**Fig. (7):**
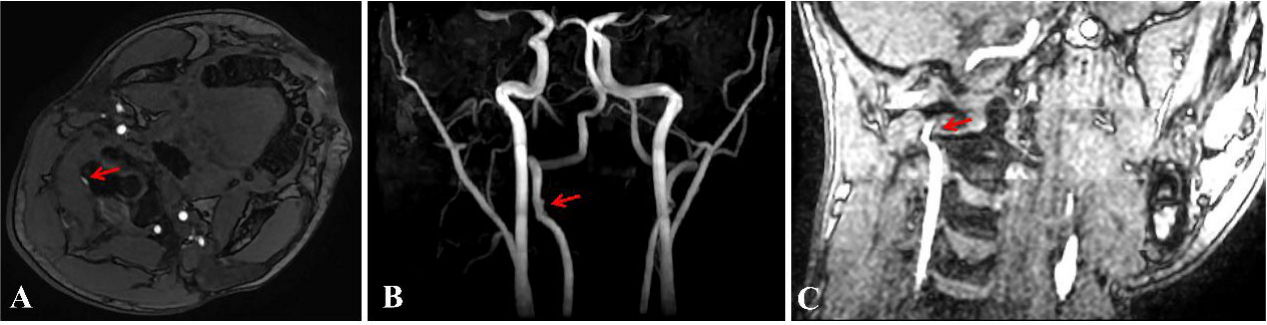
MRA with 48.96° of leftward head rotation. (**A**) Right VA (V3 segment) with localized compression; (**B**) Right VA with persistent visualization; (**C**) Right VA with a notch caused by compression from the right transverse process margin of C2.

**Fig. (8):**
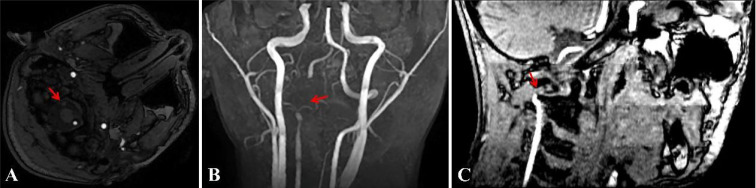
MRA with 59.69° of leftward head rotation. (**A**) Right VA (V3 segment) with faint blood-flow visualization. (**B**-**C**) Right VA with partially interrupted blood flow.

**Fig. (9):**
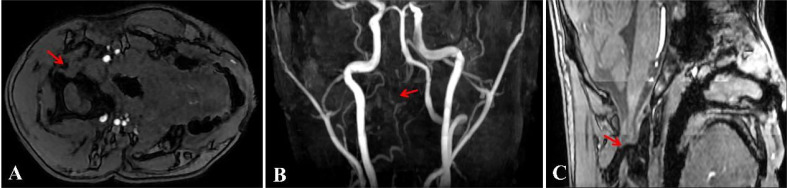
MRA with 89.19° of leftward head rotation. **(A-C**) Right VA (V4 segment) with markedly reduced blood flow, indicating complete occlusion.

**Fig. (10):**
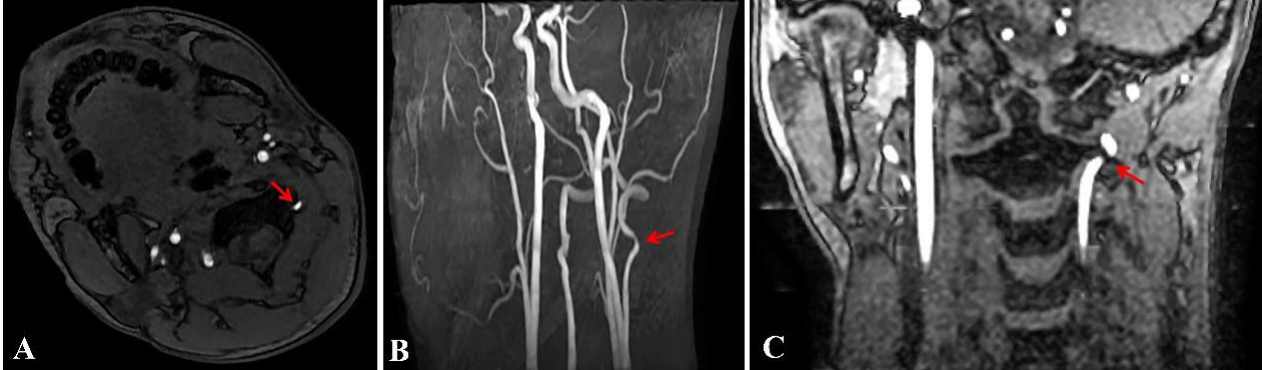
MRA with 49.25° of rightward head rotation. (**A**) Left VA (V3 segment) with localized compression; (**B**) Left VA clearly visualized; (**C**) Left VA with a notch caused by compression from the left C2 transverse-process margin.

**Fig. (11):**
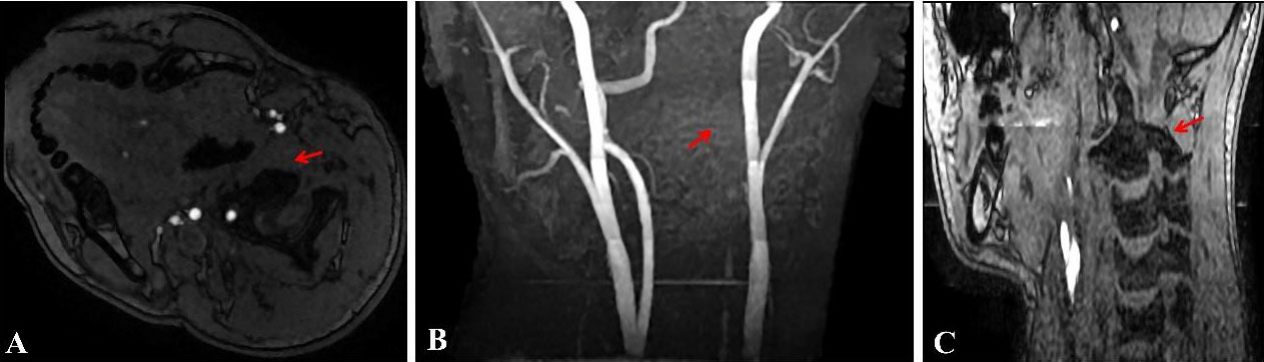
MRA with 64.94° of rightward head rotation. (**A-C**) Left VA was completely non-visualized, indicating occlusion.

On repeated physical examination, the patient reported that his dizziness had significantly worsened when turning his head to the left. Given that the patient was only 15 years old, his parents declined further DSA. To clarify the nature of the VA aneurysm and identify the cervical angles triggering dizziness, we performed high-resolution cervical vessel MRI along with dynamic cervical MRA. The findings were as follows:

- In the neutral position, bilateral VAs were visualized. The right VA (V3 segment) showed a dissecting aneurysm, with thrombosis within the false lumen, as demonstrated on high-resolution MRI (Fig. **6**).
- At 48.96° of left cervical rotation, the right VA remained visible, but focal compression by the C2 vertebra was evident at the V3 segment, showing a pronounced notch (Fig. **7**).
- At 59.69° of left rotation, flow in the right VA began to show interruption, with faint visualization of blood flow in the affected segment (Fig. **8**).
- At 89.19° of left rotation, the right VA (V4 segment) showed markedly reduced blood flow, and the remainder was not visualized, indicating complete occlusion (Fig. **9**).
- At 49.25° of right cervical rotation, the left VA was still visualized. However, the V3 segment showed localized compression by the raised margin of the C2 vertebra (Fig. **10**).
- At 64.94° of right rotation, the left VA was completely non-visualized, indicating occlusion (Fig. **11**).

Dynamic imaging showed that rotating the head approximately 50° to the left produced focal compression of the right V_3_ segment of the VA, while the flow remained patent. At approximately 90° rotation, the blood flow signal disappeared, indicating complete occlusion. Chronic mechanical compression by the sharp, elevated margin of C2 had likely caused endothelial injury in the right V3 segment, culminating in a dissecting aneurysm. Comparable changes were observed contralaterally; when the head was rotated approximately 50° to the right, the left V3 segment was focally compressed yet visible, whereas approximately 60° rotation abolished flow entirely. The pathophysiological mechanism was presumed to be identical to that on the right. The constellation of rotational vertigo, recurrent posterior-circulation infarctions, and dynamic VA flow alterations on cervical CTA and rotational MRA confirmed the diagnosis of bilateral BHS.

**Fig. (12):**
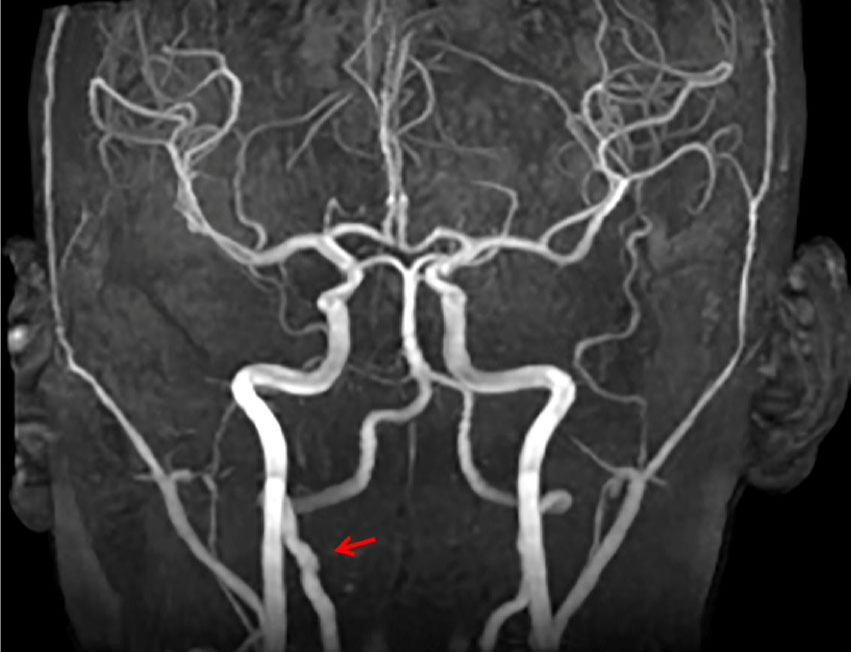
Supine-position MRA. The intramural dissecting aneurysm of the right VA at the V3 segment decreased in size compared to previous imaging.

During hospitalization, the patient was treated with aspirin (100 mg/day) and atorvastatin (20 mg/day), and a cervical collar was used to restrict excessive neck movement. At the six-month follow-up, the patient had experienced no further ischemic stroke events. Follow-up brain MRI and diffusion-weighted imaging showed no new infarctions. Supine-position MRA revealed that the intramural dissecting aneurysm of the right VA at the V3 segment had decreased in size compared to previous imaging (Fig. **12**).

## 3 DISCUSSION

In this study, we described a case of a 15-year-old boy with BHS. He had no history of cardiovascular or cerebrovascular disease. On both occasions when symptoms began, rotatory vertigo occurred after neck movement. Electrocardiography, transthoracic echocardiography, and a TCD bubble study were unremarkable. Brain MRI demonstrated multiple posterior-circulation infarcts, whereas DSA and cervical CTA indicated a right-sided vertebral-artery dissection. Dynamic cervical MRA showed normal vertebral-artery flow with the head in a neutral position, but rotation to either side resulted in interruption of flow in both VAs.

Osteophytes are the most common cause of VA occlusion [6]. However, in this study, the etiology of BHS was considered to be atlantoaxial joint instability. In pediatric cases of posterior circulation ischemic stroke, previous studies [7, 8] have reported that atlantoaxial instability can cause VA displacement during excessive cervical rotation. At certain angles, this displacement may stretch or injure the arterial endothelium, ultimately triggering thrombus formation. In the present case, the patient had no history of cervical trauma, no local osseous developmental abnormalities, and no underlying conditions, such as rheumatoid arthritis. The onset was likely related to congenital laxity or functional insufficiency of the transverse ligament of the atlas, which failed to maintain normal atlantoaxial alignment. Cervical rotation subsequently caused compression of the bilateral VAs, endothelial injury, and ultimately thrombotic occlusion.

A key feature of BHS is the interruption of VA blood flow during neck rotation, with rapid restoration when the head returns to the neutral position. Previous studies have reported that 4% of VA occlusions occur at the V1 segment, 58% at V2, 36% at V3, and 2% at V4. In patients with stenosis at the V3 or V4 segments, symptoms are more frequently triggered by contralateral head rotation, whereas stenosis at or below the V2 segment is more often associated with *ipsilateral* head rotation [9]. The degree of head rotation required to provoke BHS also varies. Some studies have reported symptom onset at rotation angles of 30°, 45°, 60°, or even 90° [10]. In the present case, both VAs were visualized in the neutral position. However, dynamic MRA demonstrated interruption of flow in the contralateral VA at the V3 segment when the head was rotated 90° to the left and approximately 60° to the right, consistent with findings in previous studies [11].

In BHS, multi-angle imaging is useful for assessing the type and mechanism of vascular compression, the severity of stenosis, and the degree of hemodynamic compromise, all of which directly influence treatment decisions. One of the hallmark characteristics of BHS is VA blood flow interruption during neck rotation, which rapidly resumes when the neck returns to a neutral position. Therefore, combining neck rotation tests with imaging examinations, such as DSA, TCD, CTA, and MRA, can improve diagnostic accuracy. CTA provides a clear visualization of vascular lesions and their relationships with surrounding structures. It also allows for multiplanar reconstructions of complex anatomy [12]. DSA enables dynamic assessment of blood flow during head rotation and is commonly used to confirm the diagnosis. Previous studies have reported that up to half of adolescent BHS cases involve bilateral vertebral arteries [3], often requiring multiple multi-angle CTA and DSA examinations for definitive diagnosis [7, 13]. This approach may expose adolescents to excessive ionizing radiation and increase the risk of contrast-induced nephropathy. Moreover, repeated or excessive neck rotation during imaging could potentially trigger posterior circulation ischemia, limiting the feasibility and acceptability of these procedures in young patients. In contrast, MRA is noninvasive and can visualize vessels distal to occlusions. It is especially beneficial for patients with significant renal insufficiency or contrast allergies who are not suitable candidates for DSA. The use of neck-rotation MRA to diagnose BHS in adolescents remains rare. Anaizi *et al*. [6] and Toluian *et al*. [14] applied neck-rotation MRA in adult cases to first identify the angle at which cervical osteophytes compressed the vertebral artery, a finding that was subsequently confirmed by DSA. However, these MRA studies employed a contrast agent. In the present case, conventional dynamic cervical MRA without contrast successfully enabled accurate diagnosis of BHS in an adolescent through multi-angle imaging, consistent with the findings reported by Matsuoka *et al*. [15]. These findings support the reliability and clinical utility of non-contrast dynamic MRA for diagnosing BHS in adolescents.

Current treatment strategies for BHS include surgical and conservative approaches, depending on the underlying pathology and clinical severity. Surgical interventions include isolated VA decompression, spinal fusion procedures, or minimally invasive endovascular techniques. Conservative management focuses on limiting excessive head and neck movement using a cervical collar, along with antiplatelet or anticoagulant therapy to reduce thromboembolic  risk [12]. In the present case, the patient was treated conservatively with a neck brace to limit cervical motion. During six months of follow-up, no recurrent syncope or ischemic events occurred. Follow-up MRA demonstrated gradual resolution of the vertebral artery dissection. These findings suggest that conservative management may be effective in selected cases of BHS.

The main limitation of this report is the relatively short follow-up period, which prevents an accurate assessment of the long-term outcomes and prognosis. Longer follow-up and additional cases are required to determine whether these findings are generalizable.

## CONCLUSION

This study is the first to demonstrate, using dynamic MRA, that BHS was the underlying cause of recurrent posterior circulation infarctions in a 15-year-old patient. It also verified the favorable mid-term efficacy of conservative management using a cervical collar. Although dynamic DSA remains the diagnostic gold standard, our findings highlight the value of dynamic MRA in diagnosing BHS, particularly for assessing dynamic vascular occlusion and the evolution of arterial dissection. Therefore, in patients, especially younger individuals, who present with posterior circulation ischemic symptoms induced by specific head or neck movements but have negative findings on routine examinations, dynamic MRA could be considered an important screening option.

## Data Availability

All data generated or analyzed during this study are included in this published article.

## LIST OF ABBREVIATIONS

BHS: Bow Hunter’s Syndrome
VA: Vertebral artery
DSA: Digital subtraction angiography
MRA: Magnetic resonance angiography
TCD: Transcranial Doppler ultrasound
MRI: Magnetic resonance imaging
CTA: Computed tomography angiography

## STANDARDS OF REPORTING

CARE guidelines were followed.
